# Changes of Small Non-coding RNAs by Severe Acute Respiratory Syndrome Coronavirus 2 Infection

**DOI:** 10.3389/fmolb.2022.821137

**Published:** 2022-02-23

**Authors:** Wenzhe Wu, Eun-Jin Choi, Binbin Wang, Ke Zhang, Awadalkareem Adam, Gengming Huang, Leo Tunkle, Philip Huang, Rohit Goru, Isabella Imirowicz, Leanne Henry, Inhan Lee, Jianli Dong, Tian Wang, Xiaoyong Bao

**Affiliations:** ^1^ Department of Pediatrics, The University of Texas Medical Branch, Galveston, TX, United States; ^2^ Department of Microbiology and Immunology, The University of Texas Medical Branch, Galveston, TX, United States; ^3^ Department of Pathology, The University of Texas Medical Branch, Galveston, TX, United States; ^4^ miRcore, Ann Arbor, MI, United States; ^5^ Department of Nuclear Engineering and Radiological Sience, University of Michigan, Ann Arbor, MI, United States; ^6^ Department of Computer Science, University of Michigan, Ann Arbor, MI, United States; ^7^ Department of Molecular, Cellular and Developmental Biology, University of Michigan, Ann Arbor, MI, United States; ^8^ The Institute for Human Infections and Immunity, The University of Texas Medical Branch, Galveston, TX, United States; ^9^ The Institute of Translational Sciences, The University of Texas Medical Branch, Galveston, TX, United States

**Keywords:** SARS-CoV-2, TRF, SARS-CoV-2-derived sncRNAs, tRF5DC, viral replication and SARS-CoV-2-derived sncRNAs

## Abstract

The ongoing pandemic of coronavirus disease 2019 (COVID-19), which results from the rapid spread of the severe acute respiratory syndrome coronavirus 2 (SARS-CoV-2), is a significant global public health threat, with molecular mechanisms underlying its pathogenesis largely unknown. In the context of viral infections, small non-coding RNAs (sncRNAs) are known to play important roles in regulating the host responses, viral replication, and host-virus interaction. Compared with other subfamilies of sncRNAs, including microRNAs (miRNAs) and Piwi-interacting RNAs (piRNAs), tRNA-derived RNA fragments (tRFs) are relatively new and emerge as a significant regulator of host-virus interactions. Using T4 PNK‐RNA‐seq, a modified next-generation sequencing (NGS), we found that sncRNA profiles in human nasopharyngeal swabs (NPS) samples are significantly impacted by SARS-CoV-2. Among impacted sncRNAs, tRFs are the most significantly affected and most of them are derived from the 5′-end of tRNAs (tRF5). Such a change was also observed in SARS-CoV-2-infected airway epithelial cells. In addition to host-derived ncRNAs, we also identified several small virus-derived ncRNAs (svRNAs), among which a svRNA derived from CoV2 genomic site 346 to 382 (sv-CoV2-346) has the highest expression. The induction of both tRFs and sv-CoV2-346 has not been reported previously, as the lack of the 3′-OH ends of these sncRNAs prevents them to be detected by routine NGS. In summary, our studies demonstrated the involvement of tRFs in COVID-19 and revealed new CoV2 svRNAs.

## Introduction

Severe acute respiratory syndrome coronavirus 2 (SARS-CoV-2) is a beta coronavirus belonging to the sarbecovirus subgenus of Coronaviridae family (Zhu et al., 2020). It is a positive-sense single-stranded RNA virus with a genome length of ∼30 kb. By the middle of January 2022, the ongoing coronavirus disease 2019 (COVID-19) pandemic, caused by SARS-CoV-2, has respectively caused more than 320 million infectious cases and over five million deaths globally (World Health, 2021).

Small non-coding RNAs (sncRNAs) have diverse functions through various regulatory mechanisms. They virtually participate in all biological pathways, including cell proliferation, differentiation, apoptosis, autophagy, and tissue remodeling. sncRNAs are also essential to regulate host responses to viral infections (Choudhuri, 2010; Beermann et al., 2016; Romano et al., 2017; Rajput et al., 2020; Wu et al., 2020). Among sncRNAs, the most widely studied sncRNAs are microRNAs (miRNAs), which are 18–24 nt in length, carry 5′ monophosphate and 3′ hydroxyl (3′-OH) ends, and generally regulate genes *via* the argonaute (AGO) platform (Schwarz et al., 2004; Fabian and Sonenberg, 2012).

Other than miRNAs, piwi-interacting RNAs (piRNAs), small nucleolar RNAs (snoRNAs), and tRNA-derived RNA fragments (tRFs) are also important members of sncRNAs (Dozmorov et al., 2013). Currently, there is very limited information on whether or how SARS-CoV-2 regulates the sncRNA expression, except the reports on SARS-CoV-2-impacted miRNAs (Mallick et al., 2009; Hasan et al., 2014; Khan et al., 2020).

Using T4 polynucleotide kinase (T4 PNK)‐RNA‐seq, a modified next-generation sequencing (NGS), we found that tRFs and piRNAs were the two most abundant sncRNAs in nasopharyngeal swabs (NPS) samples of the SARS-CoV-2-positive group. However, only tRFs were significantly enhanced in SARS-CoV positive samples. Generally, tRFs are generated by specific cleavages within pre-tRNAs or mature tRNAs (Lee et al., 2009). Compared with other sncRNAs, tRFs are relatively new members. However, their importance in diseases, such as cancer, infectious diseases, neurodegenerative diseases, and metabolic diseases, was quickly acknowledged after the discovery (Wang et al., 2013; Selitsky et al., 2015; Shen et al., 2018; Sun et al., 2018; Zhu et al., 2018; Choi et al., 2020; Qin et al., 2020; Wu et al., 2021). tRFs are classified mainly into tRF-1 series, tRF-3 series, and tRF-5 series (Fu et al., 2009). tRF-1 series are usually those from the 3′-trailer sequences of pre-tRNA, while tRF-3 and tRF-5 series are aligned to the 3′- and 5′- end of the mature tRNAs respectively. Among SARS-CoV-2-impacted tRFs, the most impacted tRFs belonged to tRF5s. In addition, the impacted tRF profile seemed to be SARS-CoV-2 specific, which is consistent with what we and others found previously on the changes in tRF signatures being virus-dependent (Wang et al., 2013; Selitsky et al., 2015), implicating tRFs as potential prognosis and diagnosis biomarkers. The impacted tRFs were also observed in SARS-CoV-2 infected human alveolar type II-like epithelial cells expressing human angiotensin-converting enzyme 2 (A549-ACE2) and human small airway epithelial cells (SAECs) in the air-liquid interface (ALI) culture.

In addition to host-derived ncRNAs, viral genomes can also encode ncRNAs. These viral ncRNAs vary in length and have diverse biological functions, including the regulation of viral replication, viral persistence, host immune evasion, host inflammatory response, and cell transformation (Tycowski et al., 2015). For example, SARS-CoV-encoded small RNAs contribute to SARS-CoV-induced lung injury (Morales et al., 2017), and SARS-CoV-2-encoded miRNAs enhance inflammation (Cheng et al., 2021). In this study, we revealed several new small viral RNA (svRNA) fragments, with the length of 25 nt, 33 nt, and 36 nt, by T4 PNK‐RNA‐seq. Among svRNAs derived from CoV-2 (sv-CoV2), a svRNA spanning from site 346 to site 382 of nsp1(sv-CoV2-346) had the highest expression.

In summary, this is the first report demonstrating the altered tRFs by SARS-CoV-2. T4 PNK pretreatment also enabled small RNA seq to reveal additional new sv-CoV2. In the future, we will characterize the biogenesis and function mechanisms of these new sncRNAs associated with SARS-CoV-2 infection.

## Materials and Methods

### Nasopharyngeal Swab Specimens

NPS were collected from patients who visited outpatient clinics of the University of Texas Medical Branch (UTMB) for SARS-CoV-2 screening in April 2020. NPS samples in universal viral transport media were transported to the Molecular Pathology laboratory, directed by Dr. Jianli Dong, and subjected to SARS-CoV-2 test using Abbott m2000 SARS-CoV-2 RT-PCR assay. The limit of detection (LOD) of detection assays is 100 viral genome copies/ml.

Thirteen anonymous NPS samples were used in this study, including seven SARS-CoV-2 negative (51.7 ± 13.7 years old) and six SARS-CoV-2 positive (49.2 ± 10.5 years old) samples. The protocol was approved by the Institutional Review Boards (IRB) of UTMB at Galveston, under the IRB protocol # 02-089 and 03-385.

### RNA Isolation

After the SARS-CoV-2 validation, 1 ml of NPS sample from each individual was subjected to RNA extraction using the mirVana PARIS kit (Invitrogen, MA, United States) according to the manufacturer’s protocol. At the elution step, samples were incubated on the column for 5 min at 65°C, and the RNA was eluted with 45 µL nuclease-free water. To extract RNAs from cells, TRIzol reagents (Thermo Fisher Scientific, MA, United States) were used for total RNA preparation, as described (Choi et al., 2020), followed by qRT-PCR.

### T4 PNK-RNA-seq and Data Analyses

To study whether other sncRNAs than miRNAs are impacted by SARS-CoV-2, we used T4 PNK-RNA-seq, a modified NGS, to get sncRNA profiles for samples derived from NSP or cultured cells, similarly as described in (Honda et al., 2015; Giraldez et al., 2019). A flowchart of the T4 PNK-RNA-seq is shown in Supplementary Figure S1A and data have been deposited in GEO (GSE193555). Basically, we treated sample RNAs with T4 PNK before the library construction and small RNA-seq to make the 3′-end of RNAs homogenous with -OH, as the ligation of the 3′-end of sncRNAs with sequencing barcodes requires the presence of 3′-OH and not all sncRNAs have 3-OH ends. The seq was done in the NGS Core of UTMB. In brief, the RNA samples were pretreated with 10 units of T4 PNK using 14 µL extracted RNAs in a final reaction volume of 50 µL and incubated at 37°C for 30 min, and then were heat-inactivated at 65°C for 20 min. The RNA was purified and concentrated within 6 µL nuclease-free water using Zymo RNA Clean and Concentrator kit (Irvine, CA, United States). Ligation-based small RNA libraries were prepared with an RNA input of 6 µL using NEB Next Multiplex Small RNA Library Prep Set for Illumina (Ipswich, MA, United States). Libraries were sequenced using the Illumina NextSeq 550 Mid-Output sequencing run. About 7,680 Mb of sequence data was generated.

To analyze the seq data, adaptor sequences were first removed using Cutadpat and reads with a length of more than 15 bp were extracted. We further filtered out RNAs with counts of less than 10 and all rRNA sequences, using the remainders as cleaned input reads. In terms of the mapping databases, we prepared tRF5 and tRF3 databases using the same sequences derived from different tRNAs [sequences downloaded from tRNA genes using the Table Browser of the UCSC genome browser (Karolchik et al., 2004)]. We also prepared tRF1 sequences using genome locations of tRNAs. Our in-house small RNA database includes 1) these tRFs, 2) miR/snoR sequences downloaded from the UCSC genome browser, and 3) piRNA sequences downloaded from piRBase (http://www.regulatoryrna.org/database/piRNA/). The cleaned input reads were mapped to our inhouse small RNA database using bowtie2 (v2.4.1) allowing two mismatches (option N -1). After we mapped the cleaned input reads to the small RNA database, the unmapped sequences were then mapped to the hg38 genome using the bowtie2 pre-built index (GRCh38_noalt_as) to detect all human sequences. The unmapped sequences to the human genome were then mapped to the SARS-CoV-2 reference genome (NC_045512) using the same parameters.

Raw read counts were normalized with the DEseq2 median of ratios method. Differentially expressed genes were determined by *p*-value < 0.05, fold change >2, and mean of normalized counts >10 in either Control (CN) or SARS-CoV-2 group. Unsupervised hierarchical clustering was performed using the Pearson correlation coefficient. A flowchart of the sequencing data analyses is summarized in Supplementary Figure S1B.

### Cell Culture and Viruses

African green monkey kidney epithelial cells (Vero E6) were obtained from ATCC (Manassas, VA, United States) and maintained in a high-glucose DMEM (Gibco, MA, United States) supplemented with 10% fetal bovine serum (FBS), 10 units/ml penicillin, and 10 μg/ml streptomycin. The human alveolar type II-like epithelial cells expressing human angiotensin-converting enzyme 2 (A549-ACE2) cells were a kind gift from Dr. Shinji Makino and were cultured in DMEM (Gibco, MA, United States) containing 10% FBS, 10 units/ml penicillin, and 10 μg/ml streptomycin.

Small airway epithelial cells (SAECs), isolated from the normal distal portion of the lung in the 1 mm bronchiole area, were purchased from Lonza (Basel, Switzerland) to generate cells in the air-liquid interface (ALI) culture. The cells were cultured and differentiated using Complete PneumaCult™-Ex plus medium and PneumaCult™-ALI-S Maintenance medium (Stemcell Technologies, Vancouver, Canada), respectively, according to the manufacturer’s instructions.

Briefly, the cells at passage two (P2) were expanded in the T-25 flask using the complete PneumaCultTM-Ex plus medium, with a medium change every other day. For ALI cultures, the cells (P3) were seeded into Corning Costar 12 mm transwell inserts (Corning, NY, United States) at a concentration of 11 × 10^4^ cells/insert in 0.5 ml medium/insert, and another 1 ml/well medium was added to the basal chamber. Cells were submerged cultured in Complete PneumaCultTM-Ex plus medium, with a medium change every other day. After reaching ∼100% confluency, ALI was initiated by removing the apical medium and replacing the PneumaCultTM-Ex plus medium in the basal compartment with PneumaCult™-ALI-S Maintenance medium. The basal compartment medium was changed every other day. It took about 21 days to complete cell differentiation.

SARS-CoV-2 (USA-WA1/2020 strain) was obtained from the World Reference Center for Emerging Viruses and Arboviruses (WRCEVA) at the UTMB. Viral stocks were prepared by propagation in Vero E6 cells. Viral titers were determined by plaque assay as described (Blanco-Melo et al., 2020). All experiments using live SARS-CoV-2 were performed in a biosafety level 3 (BSL-3) laboratory with redundant fans in the biosafety cabinets. All personnel wore powered air-purifying respirators (Breathe Easy, 3M) with Tyvek suits, aprons, booties, and double gloves. All cell cultures, cell lines or primary cultured cells, and viruses have been approved for use by the Institutional Biosafety Committee of UTMB (NOU# 2018056 and NOU# 2020043).

### Viral Infection

To infect A549-ACE2 cells in monolayer culture, the cells were seeded into the 24-well plate 24 h prior to the infection to allow the cells to reach 80–90% confluence in the following day. For infection, the cells were incubated with viruses in DMEM media with 10% FBS at a multiplicity of infection of 0.1 (MOI = 0.1). After 1 h incubation, cells were washed with PBS three times to remove the remaining viruses and cultured in fresh media containing 10% FBS. The cells were collected on day 4 post-infection.

Regarding the infection of SAECs in ALI culture, the infection was performed when hallmarks of excellent differentiation were evident, such as extensive apical coverage with cilia. Prior to infection, the apical side of the cells was washed five times with PBS, and the basal surface was washed once with PBS. Viruses were diluted to the specified MOI in 200 µL MEM medium and inoculated onto the apical surface of the ALI cultures. After a 2-h incubation at 37°C with 5% CO_2_, unbound viruses were removed by washing the surface with PBS three times. The cells were collected on day 1 or 3 post-infection. The SARS-CoV-2 S gene was detected using qRT-PCR with primers as follows: S forward primer, 5′ CCT​ACT​AAA​TTA​AAT​GAT​CTC​TGC​TTT​ACT; reverse primer, 5′’ CAA​GCT​ATA​ACG​CAG​CCT​GTA.

### qRT-PCR and RT-PCR

**FIGURE 1:**
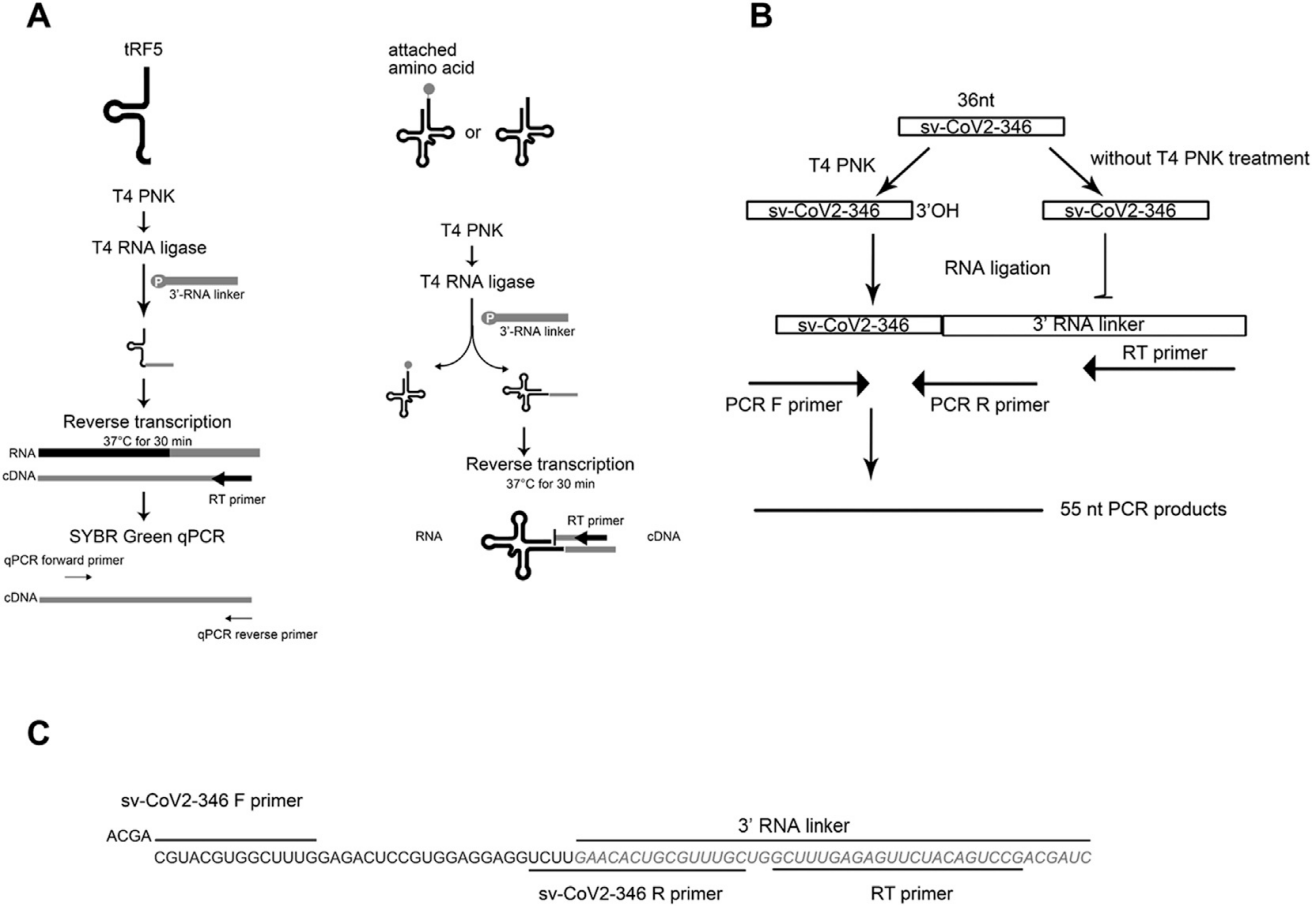
The schematic summaries on tRF quantification by qRT-PCR **(A)** and the detection of sv-CoV2-346 by RT-PCR **(B)**. **(C)** The sequence information on sv-CoV2-346 and associated primers and the 3′ RNA linker for its detection.

To evaluate sncRNAs expression, qRT-PCR was performed, as described previously (Choi et al., 2020; Wu et al., 2021). A schematic summary of tRF quantification by qRT-PCR is shown in the left panel of Figure 1A. Briefly, the total RNA was treated with T4 PNK, and then ligated to a 3′-RNA linker using T4 RNA ligase from Thermo Fisher Scientific (Waltham, MA, United States). The product was used as a template for reverse transcription (RT) with a linker-specific reverse primer using TaqMan Reverse Transcription Reagents from Thermo Fisher Scientific. The cDNA was subjected to SYBR Green qPCR using iTaq™ Universal SYBR Green Supermix kit from Bio-Rad (Hercules, CA, United States) and primers specific to the 5′-end of tRFs and RNA linker. U6 was used for normalization. The addition of a 3′-RNA linker enables the detection of tRF5s without the signal interference from its corresponding parent tRNAs, possibly because 1) the 3-end of tRNA is usually attached with an amino acid, preventing RNA linker attachment, and 2) reverse transcription annealing temperature sets tRFs, not tRNAs, to be annealed by the primer, as tRNA cloverleaf structure requires a specific denaturing temperature before annealing (right panel of Figure 1A) (Choi et al., 2020; Wu et al., 2021). The primers and 3′-RNA linker sequences are listed in Table 1.

**TABLE 1:** Sequence information for tRF5s, RNA linker, RT primer, qPCR primers and PCR primers.

tRFs	Sequence (5'-3')	
tRF5-GlyGCC	tRFs	GCA​UUG​GUG​GUU​CAG​UGG​UAG​AAU​UCU​CGC​C
	Forward primer	GCATGGGTGGTTCAGTG
	Reverse primer	CGT​CGG​ACT​GTA​GAA​CTC​TCA​AAG​C
tRF5-GluCTC	tRFs	UCC​CUG​GUG​GUC​UAG​UGG​UUA​GGA​UUC​GGC​GCU
	Forward primer	TCCCTGGTGGTCTAGTG
	Reverse primer	CGT​CGG​ACT​GTA​GAA​CTC​TCA​AAG​C
tRF5-LysCTT	tRFs	GCC​CGG​CUA​GCU​CAG​UCG​GUA​GAG​CAU​GAG​ACU
	Forward primer	GCC​CGG​CTA​GCT​CAG​TCG​GTA​G
	Reverse primer	CGT​CGG​ACT​GTA​GAA​CTC​TCA​AAG​C
tRF5-ValCAC	tRFs	GUU​UCC​GUA​GUG​UAG​UGG​UUA​UCA​CGU​UCG​CU
	Forward primer	GTT​TCC​GTA​GTG​TAG​TGG​TTA​TC
	Reverse primer	CGT​CGG​ACT​GTA​GAA​CTC​TCA​AAG​C
tRF5-CysGCA	tRFs	GGG​UAU​AGC​UCA​GUG​GUA​GAG​CAU​UUG​ACU​GC
	Forward primer	AGT​GGT​AGA​GCA​TTT​GAC​TGC
	Reverse primer	CGT​CGG​ACT​GTA​GAA​CTC​TCA​AAG​C
tRF5-GlnCTG	tRFs	UGG​UGU​AAU​AGG​UAG​CAC​AGA​GAA​UUC​UGG
	Forward primer	GGT​GTA​ATA​GGT​AGC​ACA​GAG
	Reverse primer	CGT​CGG​ACT​GTA​GAA​CTC​TCA​AAG​C
5sRNA	Forward primer	GGG​AAT​ACC​GGG​TGC​TGT​AGG
	Reverse primer	CGT​CGG​ACT​GTA​GAA​CTC​TCA​AAG​C
U6	Forward primer	GAT​GAC​ACG​CAA​ATT​CGT​GAA​GCG
	Reverse primer	CGT​CGG​ACT​GTA​GAA​CTC​TCA​AAG​C
3'RNA linker		/5Phos/GAACACUGCGUUUGCUGGCUUUGAGAGUUCUACAGUCCGACGAUC/3ddC/
RT primer		CGT​CGG​ACT​GTA​GAA​CTC​TCA​AAG​C
CoV2-346	CoV2-346	CGU​ACG​UGG​CUU​UGG​AGA​CUC​CGU​GGA​GGA​GGU​CUU
	RT primer	CGT​CGG​ACT​GTA​GAA​CTC​TCA​AAG​C
	Forward primer	ACGACGTACGTGGCTTTG
	Reverse primer	GCA​AAC​GCA​GTG​TTC​AAG​A

To validate the seq data of CoV2-encoded small RNAs (CoV2-346), RT-PCR was performed, using RT and PCR primers listed in Table 1. The overall experimental design to detect CoV2-346 by RT-PCR is illustrated in Figure 1B, with detailed seq information of primers and the RNA linker shown in Figure 1C. In brief, RNAs, pretreated with or without T4 PNK, were ligated to a 3′-RNA linker. The RT was done using primers complementary to the RNA linker, followed by PCR using forward primers annealing to 5′-end of CoV2-346 and reverse primers annealing to the last 4 nt of CoV2-346 and the first 15 nt of RNA linker.

### Northern Blot

Northern hybridization for tRF5-GluCTC was performed as described (Wang et al., 2013). Briefly, 3 µg RNA was loaded on 15% denaturing polyacrylamide gel with 7 M ureas and then transferred to a positively charged nylon membrane (Amersham Biosciences, NJ, United States). The membrane was hybridized with a ^32^P-labeled DNA probe in ULTRAhyb-Oligo solution (Life Technologies, Grand Island, NY, United States), followed by membrane washing and image development. The ^32^P-labeled DNA probe for tRF5-GluCTC was 5′-CGC​CGA​ATC​CTA​ACC​ACT​AGA​CCA​CCA-3′.

### Statistical Analysis

The experimental results were analyzed using Graphpad Prism 5 software. To compare the sncRNAs expression of NPS between SARS-CoV-2 negative and positive groups, an unpaired two-tailed Mann-Whitney *U* test was used. To compare the sncRNAs expression in SARS-CoV-2 infected cells and mock-infected cells, an unpaired two-tailed *t*-test was employed. A *p*-value < 0.05 was considered to indicate a statistically significant difference. Single and two asterisks represent a *p*-value of <0.05 and <0.01, respectively. Means ± standard errors (SE) are shown.

## Results

### T4 PNK-RNA-seq Revealed SARS-CoV-2-Impacted sncRNAs in NPS Samples.

**FIGURE 2:**
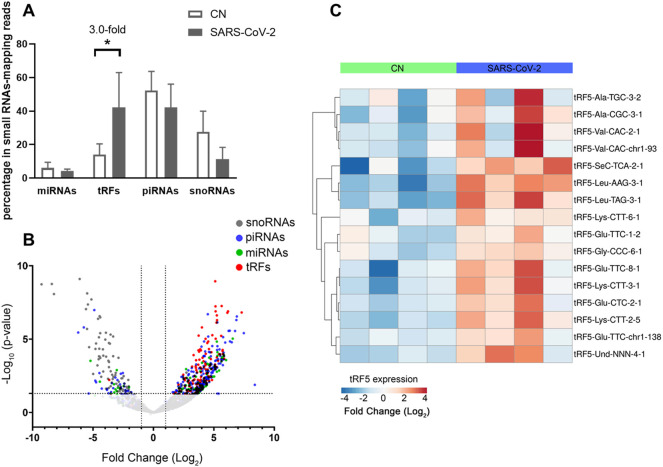
Impacted sncRNAs by SARS-CoV-2 infection in patients. **(A)** The relative sequencing frequency of miRNAs, tRFs, piRNAs, and snoRNAs was calculated by normalizing their raw reads with the DEseq2 median ratio method. **(B)** The volcano plot showed that sncRNAs were differentially expressed between and control group (CN) and SARS-CoV-2 patient group (SARS-COV-2). **(C)** Heatmap for unsupervised clustering of the differently expressed tRFs with >20 mean of normalized counts in any groups according to Pearson correlation. Data are shown as means ± standard error (SE). The single asterisk represents *p* values of <0.05.

To identify SARS-CoV-2-impacted sncRNAs, we initialized T4 PNK-RNA-seq for the NPS samples from four SARS-CoV-2 positive patients with their ages at 54.3 ± 4.0 years old and four SARS-CoV-2 negative subjects, with matched age at 50.5 ± 10.2 years old. The seq data were analyzed similarly as described in (Wang et al., 2013; Liu et al., 2018). In brief, the sequences with length >15 bp and reads >10 were mapped to the in-house small RNA database containing tRFs, miR/snoRs, and piRs to address redundant tRNA sequences across the genome after removing rRNAs. Unmapped sequences were then mapped to the hg38 human genome to identify other human-derived sequences and their composition. We found that piRNAs and tRFs were the two most abundant sncRNAs in SARS-CoV-2 positive samples. The top-10 ranked piRNAs and tRFs in the SARS-CoV-2 positive group are listed in Supplementary Tables S1, S2, respectively. As shown in Supplementary Table S2, all tRFs were derived from the 5′-ends of tRNAs, therefore tRF5s. Compared with the tRFs and piRNAs, the overall reads of miRNAs were much less (Figure 2A). We also compared the sncRNA profiles between SARS-CoV-2 positive and negative samples. As shown in Figure 2A, while the tRFs consisted of about 14% of all sncRNA counts in the control group, tRFs counts became 42% in the COVID-19 group, demonstrating a significant increase by COVID-19. In contrast, the overall expression of miRNAs and piRNAs was not impacted by COVID-19 (Figure 2A).

Differential expression analysis for individual sncRNAs was also performed for SARS-CoV-2 negative and positive groups. As shown in Figure 2B, there were more up-regulated tRFs than down-regulated tRFs, while SARS-CoV-2 down-regulated snoRNAs were more than up-regulated ones. We also listed sncRNAs, which were significantly altered by SARS-CoV-2 in Tables 2–4. The cutoff was set as a fold change >2, with the significance of *p* < 0.05 in changes by SARS-CoV-2, and the mean of normalized counts >10 in the negative or positive group. The differentially expressed tRFs, miRNAs, and snoRNAs were listed in Tables 2–4, respectively.

**TABLE 2:** Changes in tRFs by SARS-CoV-2.

	Mean		Log2FC	Fold Change (FC)	*p*-value	Padj
	CN	COVID-19				
Down-regulated						
tRF1_chr5_24_Lys_CTT_26198538	19.94	1.62	−3.65	12.54	0.00607	0.02862
Up-regulated						
tRF5-Val-TAC-1-2	1.43	32.82	4.31	19.79	0.00047	0.00439
tRF5-Val-CAC-chr1-93	65.75	869.52	3.72	13.20	0.00056	0.00498
tRF5-Val-CAC-3-1	3.38	27.07	2.86	7.26	0.00781	0.03457
tRF5-Val-CAC-2-1	6.11	120.86	4.34	20.25	0.00010	0.00151
tRF5-Und-NNN-4-1	9.13	128.84	3.79	13.85	0.00007	0.00117
tRF5-Thr-CGT-6-1	5.28	95.45	4.08	16.86	0.00019	0.00220
tRF5-Ser-CGA-2-1	0.92	10.74	4.81	28.14	0.00112	0.00798
tRF5-SeC-TCA-2-1	41.06	609.64	3.94	15.34	0.00006	0.00099
tRF5-nmt-Gln-TTG-6-1	0.39	21.22	5.23	37.52	0.00079	0.00632
tRF5-Lys-CTT-chr15-5	0.45	14.56	4.60	24.24	0.00389	0.02083
tRF5-Lys-CTT-6-1	27.07	118.09	2.09	4.27	0.01283	0.04915
tRF5-Lys-CTT-3-1	13.01	199.40	3.89	14.80	0.00005	0.00089
tRF5-Lys-CTT-2-5	40.64	667.89	4.03	16.29	0.00000	0.00006
tRF5-Leu-TAG-3-1	2.23	73.96	5.23	37.54	0.00000	0.00003
tRF5-Leu-CAG-2-1	2.55	32.99	3.57	11.84	0.00132	0.00913
tRF5-Leu-AAG-3-1	7.52	247.62	5.12	34.87	0.00000	0.00000
tRF5-iMet-CAT-1-8	3.33	109.22	4.89	29.56	0.00001	0.00017
tRF5-His-GTG-2-1	3.93	34.02	3.00	8.03	0.01555	0.05736
tRF5-Gly-CCC-6-1	4.71	20.69	2.14	4.39	0.02402	0.07725
tRF5-Glu-TTC-chr1-138	82.66	458.76	2.48	5.59	0.00270	0.01595
tRF5-Glu-TTC-8-1	140.13	1637.50	3.54	11.63	0.00028	0.00287
tRF5-Glu-TTC-1-2	13.42	47.61	1.85	3.60	0.04498	0.12120
tRF5-Glu-CTC-3-1	1.18	12.02	4.17	18.05	0.00619	0.02892
tRF5-Glu-CTC-2-1	8234.45	83040.94	3.33	10.08	0.00012	0.00173
tRF5-Ala-TGC-3-2	3.89	27.72	2.98	7.89	0.01629	0.05924
tRF5-Ala-CGC-3-1	2.93	29.71	3.41	10.60	0.00335	0.01915
tRF5-Ala-CGC-2-1	1.59	27.28	3.95	15.42	0.00182	0.01173
tRF5-Ala-AGC-4-1	2.44	27.91	3.41	10.62	0.00566	0.02723
tRF3-Val-TAC-3-1	1.08	49.08	5.17	35.90	0.00001	0.00024
tRF3-Val-TAC-1-2	3.11	79.25	4.47	22.16	0.00002	0.00040
tRF3-Val-CAC-chr1-93	0.72	47.24	5.62	49.09	0.00001	0.00018
tRF3-Val-AAC-4-1	1.25	43.10	4.81	28.06	0.00008	0.00122
tRF3-Trp-CCA-5-1	0.16	16.24	5.45	43.78	0.00011	0.00156
tRF3-Trp-CCA-4-1	2.46	112.29	5.26	38.29	0.00000	0.00001
tRF3-Thr-TGT-5-1	7.34	36.03	2.62	6.14	0.01642	0.05924
tRF3-Thr-CGT-4-1	1.83	14.12	2.86	7.24	0.03537	0.09972
tRF3-Thr-CGT-3-1	0.59	10.11	3.69	12.94	0.00596	0.02835
tRF3-Thr-CGT-1-1	0.00	12.25	5.76	54.09	0.00010	0.00151
tRF3-Thr-AGT-5-1	0.49	12.34	4.34	20.24	0.00116	0.00823
tRF3-Thr-AGT-4-1	0.16	12.77	5.11	34.64	0.00054	0.00485
tRF3-Thr-AGT-3-1	0.33	12.24	4.50	22.66	0.00120	0.00843
tRF3-SeC-TCA-2-1	2.95	128.28	5.28	38.72	0.00000	0.00002
tRF3-Pro-TGG-3-5	38.00	215.39	2.53	5.77	0.00515	0.02533
tRF3-Phe-GAA-10-1	4.75	119.56	4.54	23.34	0.00001	0.00033
tRF3-nm-Tyr-GTA-chr21-2	2.28	12.21	2.29	4.90	0.03717	0.10322
tRF3-nmt-Gln-TTG-9-1	7.30	276.42	5.16	35.76	0.00000	0.00001
tRF3-nmt-Gln-TTG-7-1	7.17	278.12	5.15	35.42	0.00000	0.00001
tRF3-Lys-TTT-14-1	4.97	73.70	3.84	14.29	0.00686	0.03108
tRF3-Lys-CTT-9-1	0.00	10.54	5.54	46.55	0.00011	0.00163
tRF3-Leu-TAG-3-1	6.59	143.71	4.32	20.02	0.00001	0.00024
tRF3-Leu-TAG-2-1	15.06	196.02	3.74	13.35	0.00006	0.00099
tRF3-Leu-TAG-1-1	3.46	72.59	4.61	24.39	0.00005	0.00089
tRF3-Leu-CAG-chr9-7	6.77	50.14	2.95	7.74	0.01033	0.04250
tRF3-Leu-CAG-1-6	7.52	51.42	2.93	7.64	0.00670	0.03063
tRF3-Leu-AAG-7-1	2.89	45.39	3.81	14.06	0.00025	0.00271
tRF3-Leu-AAG-2-4	14.00	184.25	3.75	13.46	0.00001	0.00017
tRF3-iMet-CAT-1-6	0.52	15.97	4.67	25.41	0.00067	0.00567
tRF3-Gly-TCC-2-5	1.86	201.79	6.53	92.33	0.00000	0.00000
tRF3-Gly-CCC-7-1	14.44	134.89	3.22	9.29	0.00075	0.00611
tRF3-Gln-TTG-3-3	1.08	12.93	3.26	9.57	0.00691	0.03113
tRF3-Gln-CTG-5-1	0.82	12.55	3.63	12.37	0.00186	0.01193
tRF3-Gln-CTG-1-5	0.23	19.81	5.73	53.01	0.00007	0.00108
tRF3-Cys-GCA-9-4	0.33	14.30	4.70	26.06	0.00061	0.00525
tRF3-Cys-GCA-5-1	0.82	15.93	3.98	15.77	0.00106	0.00777
tRF3-Cys-GCA-4-1	0.69	16.86	4.33	20.09	0.00059	0.00514
tRF3-Cys-GCA-24-1	0.71	15.60	4.20	18.42	0.00082	0.00645
tRF3-Cys-GCA-23-1	0.62	21.73	4.77	27.20	0.00023	0.00257
tRF3-Cys-GCA-21-1	0.16	19.72	5.74	53.58	0.00006	0.00095
tRF3-Cys-GCA-17-1	1.10	19.03	3.89	14.79	0.00084	0.00657
tRF3-Cys-GCA-10-1	0.26	14.50	5.26	38.21	0.00018	0.00211
tRF3-Arg-TCT-1-1	2.49	118.78	5.66	50.51	0.00000	0.00001
tRF3-Arg-CCT-5-1	1.58	12.23	2.77	6.84	0.03258	0.09412
tRF3-Arg-CCT-4-1	2.60	13.48	2.19	4.57	0.03323	0.09521
tRF3-Arg-CCG-2-1	4.96	61.43	3.51	11.36	0.00072	0.00595
tRF3-Ala-TGC-4-1	1.46	30.32	4.16	17.91	0.00033	0.00320
tRF3-Ala-TGC-3-1	3.57	44.92	3.53	11.52	0.00091	0.00693
tRF3-Ala-TGC-2-1	0.91	75.23	6.13	70.14	0.00000	0.00001
tRF3-Ala-TGC-1-1	0.16	58.71	7.31	159.08	0.00000	0.00001
tRF3-Ala-CGC-4-1	14.18	93.77	2.66	6.30	0.00456	0.02318
tRF3-Ala-AGC-4-1	0.98	80.87	6.19	72.78	0.00000	0.00001
tRF3-Ala-AGC-2-2	14.67	92.70	2.60	6.07	0.00638	0.02969
tRF1_chr2_6_Glu_TTC_75124115	0.00	13.35	5.86	58.15	0.00022	0.00249

**TABLE 3:** Changes in miRNAs by SARS-CoV-2.

	Mean		Log2FC	Fold Change (FC)	*p*-value	Padj
	CN	COVID-19				
Up-regulated						
hsa-miR-4443	1.99	497.56	8.16	286.11	3.6E-11	1.5E-08
hsa-miR-12116	0.23	36.13	6.57	95.26	9.1E-06	0.00024
hsa-miR-765	0.00	14.76	6.02	65.02	0.00027	0.00282
hsa-miR-1224-3p	0.00	13.04	5.84	57.20	0.00014	0.00181
hsa-miR-6880-3p	0.00	12.98	5.83	56.87	0.00012	0.00165
hsa-miR-6886-3p	0.00	11.95	5.71	52.37	0.00017	0.00202
hsa-miR-6758-5p	0.16	19.09	5.68	51.37	0.00019	0.0022
hsa-miR-4716-5p	0.00	11.28	5.64	49.85	0.00032	0.00317
hsa-miR-1281	0.00	10.01	5.45	43.62	0.00137	0.0093
hsa-miR-6741-3p	0.45	23.94	5.31	39.69	7.7E-05	0.00122
hsa-miR-769-5p	0.26	14.85	5.31	39.64	0.00013	0.00181
hsa-miR-877-3p	1.17	54.42	5.26	38.26	1.5E-05	0.00033
hsa-miR-1469	0.16	13.97	5.26	38.23	0.00164	0.01068
hsa-miR-10401-5p	1.58	59.29	5.07	33.70	0.00067	0.00567
hsa-miR-7111-3p	0.23	12.72	5.07	33.59	0.00073	0.00597
hsa-miR-4646-3p	0.23	10.42	4.78	27.49	0.00106	0.00777
hsa-miR-204-3p	0.66	24.52	4.73	26.59	0.0005	0.00463
hsa-miR-7107-5p	2.14	59.30	4.62	24.67	0.00018	0.00215
hsa-miR-6510-5p	0.49	17.34	4.55	23.44	0.00052	0.00473
hsa-miR-6823-3p	0.85	23.17	4.48	22.27	0.00048	0.00441
hsa-miR-3196	8.17	141.16	4.04	16.41	0.0004	0.00389
hsa-miR-665	0.78	14.73	3.89	14.79	0.00153	0.0102
hsa-miR-7847-3p	2.36	30.55	3.53	11.52	0.00358	0.01993
hsa-miR-1268b	3.52	32.94	3.05	8.30	0.00432	0.02222
hsa-miR-139-3p	1.28	12.34	3.00	7.98	0.01266	0.04865
hsa-miR-4728-5p	1.10	10.31	2.98	7.89	0.01414	0.05307
hsa-miR-320c	8.22	62.47	2.85	7.20	0.00743	0.03314
hsa-miR-92b-5p	87.47	600.46	2.77	6.80	0.00429	0.02216
hsa-miR-320b	13.69	93.88	2.71	6.56	0.00109	0.00783
hsa-miR-140-3p	1.48	11.69	2.70	6.49	0.01402	0.05278
hsa-miR-186-5p	6.64	39.95	2.46	5.50	0.02296	0.07477
hsa-miR-378a-3p	9.29	50.19	2.34	5.07	0.02429	0.07761
hsa-miR-1268a	5.61	27.61	2.15	4.43	0.02005	0.06879
hsa-miR-762	5.81	27.57	2.13	4.38	0.03574	0.09995
hsa-miR-2110	8.28	38.68	2.12	4.35	0.02247	0.07433
Down-regulated						
hsa-miR-34b-3p	43.92	1.10	−5.20	36.68	0.0003	0.00306
hsa-miR-328-3p	26.78	3.38	−2.93	7.60	0.02036	0.06968
hsa-miR-6510-3p	15.86	1.64	−3.25	9.54	0.01166	0.04608
hsa-miR-26a-5p	143.78	25.93	−2.45	5.48	0.01568	0.05743
hsa-miR-99a-5p	657.50	163.26	−2.01	4.03	0.01872	0.06548
hsa-miR-92b-3p	346.22	42.48	−3.02	8.12	0.00127	0.0089

**TABLE 4:** Changes in snoRNAs by SARS-CoV-2.

	Mean		Log2FC	Fold Change (FC)	*p*-value	Padj
	CN	COVID-19				
Down-regulated						
hg38_wgRna_ACA49	227.26	0.27	−9.25	609.86	1.9E-09	3.4E-07
hg38_wgRna_ACA40	180.98	0.53	−8.40	338.05	1.7E-09	3.4E-07
hg38_wgRna_ACA28	219.23	0.72	−8.22	299.21	8.6E-09	1.1E-06
hg38_wgRna_ACA8	409.75	8.53	−5.57	47.58	9.1E-08	7.7E-06
hg38_wgRna_U92	79.97	1.72	−5.49	44.82	2.1E-06	8E-05
hg38_wgRna_E3	162.73	3.73	−5.41	42.66	4.4E-08	4.7E-06
hg38_wgRna_ACA25	38.67	0.92	−5.31	39.75	3.6E-05	0.00069
hg38_wgRna_ACA16	158.54	4.34	−5.13	34.92	2E-08	2.3E-06
hg38_wgRna_ACA3	206.55	7.70	−4.74	26.71	2E-05	0.00042
hg38_wgRna_U73a	25.28	1.01	−4.62	24.63	0.00062	0.00531
hg38_wgRna_U35A	99.03	4.05	−4.58	23.87	4.2E-07	2.1E-05
hg38_wgRna_ACA38	35.59	1.45	−4.53	23.09	0.00024	0.00258
hg38_wgRna_mgU6-77	93.30	4.01	−4.50	22.65	7.6E-06	0.0002
hg38_wgRna_HBII-82B	36.20	1.76	−4.50	22.57	0.00015	0.00187
hg38_wgRna_E2	88.91	4.05	−4.48	22.26	4.5E-05	0.00087
hg38_wgRna_ACA63	122.12	5.51	−4.45	21.84	0.00016	0.00192
hg38_wgRna_HBII-180A	157.02	7.50	−4.39	21.04	4E-06	0.00013
hg38_wgRna_U71a	38.72	1.83	−4.39	20.95	0.00019	0.00221
hg38_wgRna_ACA41	21.63	1.00	−4.31	19.86	0.00372	0.02024
hg38_wgRna_U90	86.50	4.66	−4.18	18.07	5E-05	0.00089
hg38_wgRna_ACA10	38.56	2.01	−4.15	17.73	0.00162	0.0106
hg38_wgRna_U97	89.21	4.93	−4.14	17.68	0.00013	0.00181
hg38_wgRna_ACA44	114.67	7.12	−3.97	15.72	1.1E-05	0.00027
hg38_wgRna_ACA9	13.58	1.00	−3.75	13.48	0.00509	0.02516
hg38_wgRna_ACA51	85.91	6.42	−3.72	13.16	0.0003	0.00306
hg38_wgRna_ACA3-2	124.34	9.63	−3.68	12.78	0.00082	0.00645
hg38_wgRna_U15A	87.31	6.73	−3.67	12.73	0.00036	0.00352
hg38_wgRna_U15B	37.76	3.37	−3.53	11.57	0.00156	0.01027
hg38_wgRna_ACA1	28.02	2.69	−3.43	10.81	0.00929	0.0395
hg38_wgRna_ACA53	62.15	5.85	−3.41	10.60	0.0027	0.01595
hg38_wgRna_SNORD123	21.14	2.10	−3.36	10.30	0.00525	0.02556
hg38_wgRna_U17b	86.15	8.67	−3.32	10.02	0.00015	0.00184
hg38_wgRna_ACA6	31.12	3.13	−3.31	9.89	0.00373	0.02024
hg38_wgRna_U34	33.68	3.92	−3.15	8.87	0.00286	0.01669
hg38_wgRna_U18B	16.65	1.90	−3.07	8.38	0.01113	0.04479
hg38_wgRna_SNORA38B	13.91	1.82	−2.91	7.49	0.01566	0.05743
hg38_wgRna_U32A	70.17	9.50	−2.86	7.27	0.00813	0.03551
hg38_wgRna_U52	41.92	6.17	−2.79	6.92	0.03576	0.09995
hg38_wgRna_U51	26.73	4.02	−2.75	6.73	0.02161	0.07241
hg38_wgRna_U17a	28.75	4.49	−2.64	6.21	0.00479	0.02396
hg38_wgRna_HBII-85-18	38.90	6.61	−2.53	5.76	0.02759	0.08448
hg38_wgRna_U19-2	18.33	4.68	−2.00	4.00	0.04758	0.12608

As shown in Table 2, tRFs were significantly impacted by SARS-CoV-2 in NPS. Among those, 2 tRFs belong to the tRF1 series, 28 tRFs were tRF5s, and 53 tRFs were tRF3s. However, top-ranked SARS-CoV-2-impacted tRFs all belong to the tRF5. In Figure 2C, the mean of normalized counts >20 in the control (CN) or SARS-CoV-2 positive (SARS-CoV-2) group were selected to plot the heatmap and their sequences were listed in Table 5.

**TABLE 5:** tRF5s with mean of normalized counts > 20 in Control (CN) or COVID-19 groups.

	Mean		Log2FC	Fold Change (FC)	*p*-value	Padj	Sequence	Length (nt)
	CN	COVID-19						
Up-regulated								
tRF5-Ala-TGC-3-2	3.89	27.72	2.98	7.89	0.01629	0.05924	GGG​GAU​GUA​GCU​CAG​UGG​C	19
tRF5-Ala-CGC-3-1	2.93	29.71	3.41	10.60	0.00335	0.01915	GGGGAUGUAGCUCAGUGG	18
tRF5-Val-CAC-2-1	6.11	120.86	4.34	20.25	0.00010	0.00151	GCU​UCU​GUA​GUG​UAG​UGG​UUA​UCA​CGU​UCG​CCU​C	34
tRF5-Val-CAC-chr1-93	65.75	869.52	3.72	13.20	0.00056	0.00498	GUU​UCC​GUA​GUG​UAG​UGG​UUA​UCA​CGU​UCG​CC	32
tRF5-SeC-TCA-2-1	41.06	609.64	3.94	15.34	0.00006	0.00099	AGUGGUCUGGGGUGC	15
tRF5-Leu-AAG-3-1	7.52	247.62	5.12	34.87	0.00000	0.00000	GGUAGCGUGGCCGAGC	16
tRF5-Leu-TAG-3-1	2.23	73.96	5.23	37.54	0.00000	0.00003	GGUAGCGUGGCCGAGU	16
tRF5-Lys-CTT-6-1	27.07	118.09	2.09	4.27	0.01283	0.04915	AGC​UCA​GUC​GGU​AGA​GCA​UGG​GAC​A	25
tRF5-Glu-TTC-1-2	13.42	47.61	1.85	3.60	0.04498	0.12120	AUG​GUC​UAG​CGG​UUA​GGA​UUC​CUG​GU	26
tRF5-Gly-CCC-6-1	4.71	20.69	2.14	4.39	0.02402	0.07725	AGUGGUAGAAUUCUCGCC	18
tRF5-Glu-TTC-8-1	140.13	1637.50	3.54	11.63	0.00028	0.00287	UCC​CCU​GUG​GUC​UAG​UGG​UUA​GGA​UUC​GGC​GCU	33
tRF5-Lys-CTT-3-1	13.01	199.40	3.89	14.80	0.00005	0.00089	GCC​CGG​CUA​GCU​CAG​UCG​GUA​GAG​CAU​GAG​ACC	33
tRF5-Glu-CTC-2-1	8234.45	83040.94	3.33	10.08	0.00012	0.00173	UCC​CUG​GUG​GUC​UAG​UGG​UUA​GGA​UUC​GGC​GCU	33
tRF5-Lys-CTT-2-5	40.64	667.89	4.03	16.29	0.00000	0.00006	GCC​CGG​CUA​GCU​CAG​UCG​GUA​GAG​CAU​GAG​ACU	33
tRF5-Glu-TTC-chr1-138	82.66	458.76	2.48	5.59	0.00270	0.01595	UCC​CUG​GUG​GUC​UAG​UGG​CUA​GGA​UUC​GGC​GCU	33
tRF5-Und-NNN-4-1	9.13	128.84	3.79	13.85	0.00007	0.00117	UCC​CUG​UAG​UCU​AGU​GGU​UAG​GAU​UCG​GCG​CU	32

### Experimental Validation of SARS-CoV-2-Impacted tRFs

**FIGURE 3:**
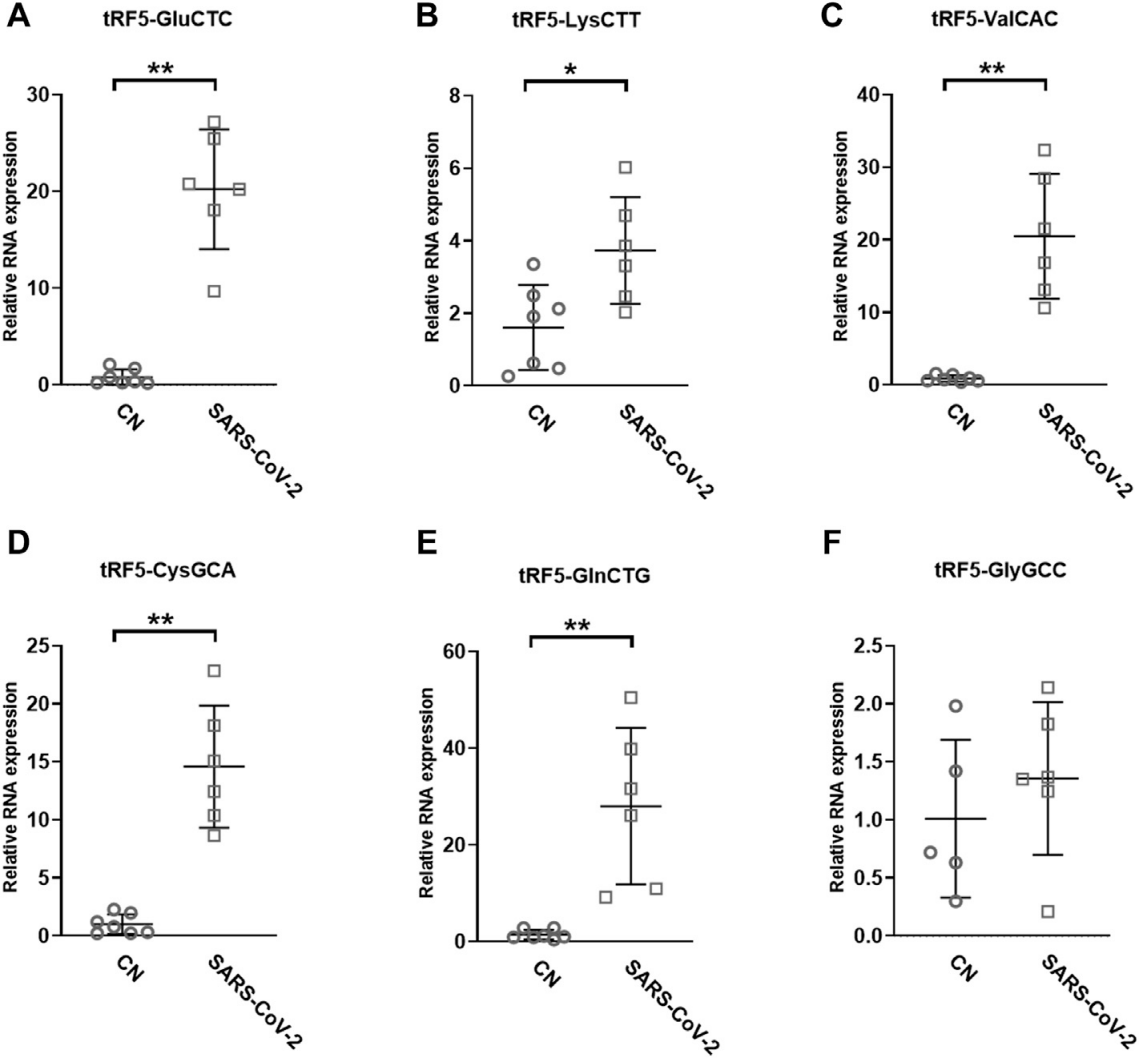
Changes in the expression of tRF5s in NPS samples by SRAS-CoV-2. qRT-PCR was performed to detect tRF5-GluCTC **(A)**, tRF5-LysCTT **(B)**, tRF5-ValCAC **(C)**, tRF5-CysGCA **(D)**, tRF5-GlnCTG **(E)**, and tRF5-GlyGCC **(F)** in the NPS from SARS-COV-2 and control (CN) patients. U6 was used as an internal control. Unpaired two-tailed Mann-Whitney U tests were performed for statistical comparisons. Single and double asterisks represent *p* values of <0.05 and <0.01, respectively. Data are shown as means ± SE.

To validate the seq data, we used modified qRT-PCR to detect the expression of tRF5-GluCTC, tRF5-LysCTT, and tRF5-ValCAC, three top-ranked tRF5s in SARS-CoV-2-positive patients according to the seq data, as we previously described (Choi et al., 2020; Wu et al., 2021). Compared with Northern blot validation, the modified qRT-PCR with T4 PNK pretreatment and 3′-end RNA linker ligation provides the possibility to validate as many tRFs as possible for NPS samples, which usually have a limited yield of RNAs. Our results demonstrated that tRF5-GluCTC, tRF5-LysCTT, and tRF5-ValCA were significantly increased in the SARS-CoV-2 group (Figures 3A–C). Unlike SARS-CoV-2, which could induce tRF5-ValCAC, respiratory syncytial virus (RSV), a negative-sense RNA virus, does not induce tRF5-ValCAC infected cells (Wang et al., 2013), suggesting the change in tRF profile in response to viral infections is virus-specific.

Other than the three tRF5s mentioned above, tRF5-CysGCA, tRF5-GlnCTG and tRF5-GlyGCC were also chosen for the validation, as these three tRFs are highly inducible by RSV with the function tRF5-GlyGCC and tRF5-GlnCTG being vital in promoting RSV replication (Choi et al., 2020; Zhou et al., 2017). Although the function of tRF5-CysGCA in RSV is not clear in viral infection, it is important in regulating stress responses and neuroprotection (Ivanov et al., 2014). We validated that tRF5-CysGCA and tRF5-GlnCTG were also significantly enhanced in the SARS-CoV-2 group, compared with the control (CN) group (Figures 3D,E). However, SARS-CoV-2 did not affect tRF5-GlyGCC expression (Figure 3F). Given the fact that RSV induces significantly tRF5-GlyGCC (Wang et al., 2013), the result of Figure 3F supported virus-specific induction of tRFs and tRFs as potential biomarkers of viral infection.

### Impacted tRFs in SARS-CoV-2-Infected Cells

**FIGURE 4:**
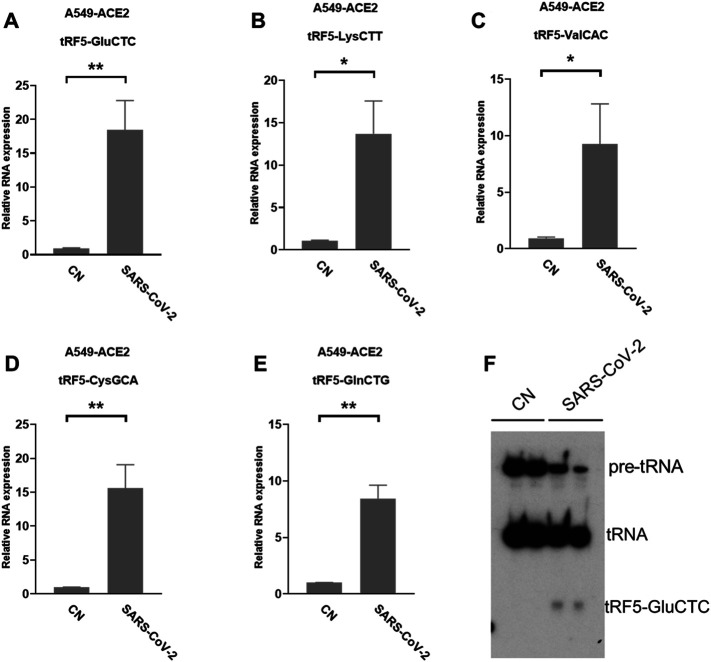
SARS-CoV-2-impacted tRF5s in A549-ACE2 cells. **(A–E)** A549-ACE2 cells were infected with SARS-CoV-2 at an MOI of 0.1 for 4 days, qRT-PCR was performed to detect tRF5-GluCTC **(A)**, tRF5-LysCTT **(B)**, tRF5-ValCAC **(C)**, tRF5-CysGCA **(D)**, and tRF5-GlnCTG **(E)**. A northern blot was performed to detect tRF5-GluCTC **(F)**. Mock-infected cells (those without SARS-CoV-2 infection) were used as control (CN). Unpaired two-tailed t-tests were performed for statistical comparisons. Single and double asterisks represent *p* values of <0.05 and <0.01, respectively. Data, shown as means ± SE, are representative of three independent experiments.

A549-ACE2 is a common cell model to study coronaviruses, such as SARS-CoV and SARS-CoV-2 (Mossel et al., 2005; Blanco-Melo et al., 2020; Weston et al., 2020; Buchrieser et al., 2021). Herein, we studied whether the induction of tRFs can be recapitulated in SARS-CoV-2-infected A549-ACE2. As shown in Figures 4A–E, A549-ACE2 cells, at day 4 post-infection (p.i.) of SARS-CoV-2, had dramatically induction of tRF5-GluCTC, tRF5-LysCTT, tRF5-ValCAC, tRF5-CysGCA, and tRF5-GlnCTG. The northern blot also confirmed the induction of tRF5-GluCTC (Figure 4F), which was the most abundant tRF5 among the tested four tRF5s, confirming the liability of our newly developed qRT-PCR.

**FIGURE 5:**
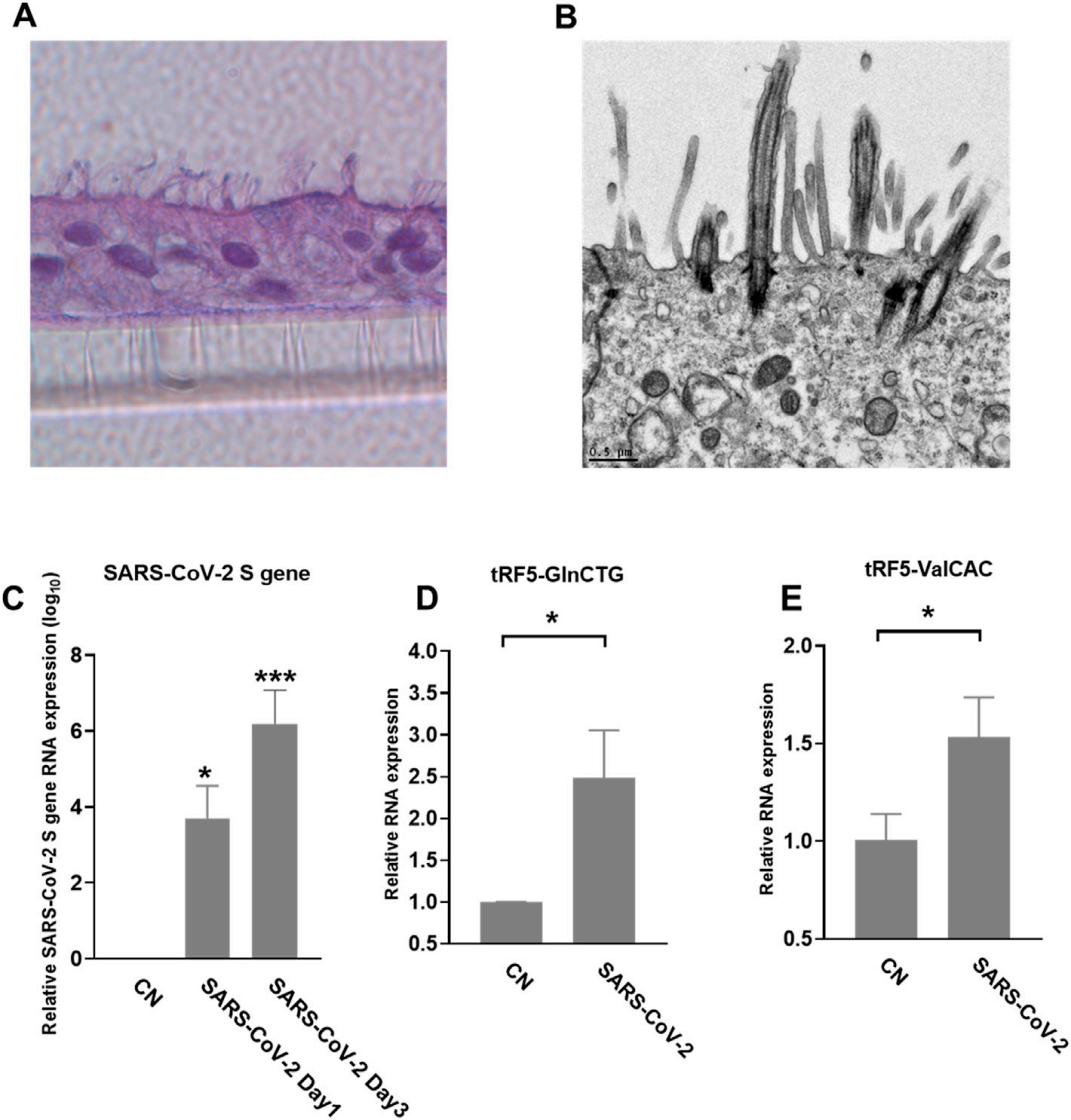
SARS-CoV-2-affected tRF5s in ALI-cultured SAECs. **(A)** Histological examination SAECs in ALI culture. After SAECs were in ALI culture for 21 days, the insert was fixed with 4% polyoxymethylene, followed by tissue processing, sectioning, and H&E staining. **(B)** Transmission electron microscopy (TEM) of SAECs in ALI culture. **(C)** ALI-cultured SEACs were apically infected with SARS-CoV-2 at an MOI of 0.1 for 1 or 3 days, qRT-PCR was performed to detect SARS-CoV-2 S gene expression. GAPDH was used as an internal control. **(D,E)** On day 3 postinfection, tRF5-GlnCTG **(D)** and tRF5-ValCAC **(E)** expressions were measured using qRT-PCR. Mock-infected cells were used as control (CN). Unpaired two-tailed t-tests were performed for statistical comparisons. Single asterisks represent *p* values of <0.05. Data are shown as means ± SE and are representative of three independent experiments.

We also used primary SAECs in ALI culture, a commonly acknowledged physiology airway infection model (Bhowmick and Gappa-Fahlenkamp, 2016; Chandorkar et al., 2017), to confirm SARS-CoV-2-affected tRFs. SAECs, after a few weeks of ALI culture, have been shown to establish a pseudostratified cell layer that resembles the small airway epithelium as found *in vivo* (Hiemstra et al., 2018). Moreover, SAECs in ALI cultures have been found to express the receptor for SARS-CoV-2, therefore, a physiologically relevant cell model to study SARS-CoV-2 (Zhang et al., 2020; Zhu et al., 2020; Schweitzer et al., 2021). Therefore, we also studied tRF5s expression in SAECs in ALI culture. As shown in Figures 5A,B, the cilia were oriented towards the upper transwell compartment, after the cells were in ALI culture for 21 days. The differentiated cultures were infected with SARS-CoV-2 at an MOI of 0.1 for 1 or 3 days, followed by viral S gene quantification using qRT-PCR (Figure 5C). Our qRT-PCR confirmed the expression change in tRF5-GlnCTG and tRF5-ValCAC, two tRFs with relatively low abundance in SARS-CoV-2 positive NPS samples and infected A549-ACE2 cells, can also be detected in SAECs in ALI culture (Figures 5D, E). Since the cleaved tRNAs account for a very tiny portion of parent tRNAs, the difference in the induction folds of tRF5-GlnCTG and tRF5-ValCAC should not be determined by the abundance of their parent tRNAs, but possibly resulted from the distinct sensitivities of their parent tRNAs to the cleavage during the infection. Overall, in this study, we established two cell models, A549-AEC2 cells in monolayer culture and SAECs in ALI culture, to characterize SARS-CoV-2-induced tRFs in the future.

### Identification of SARS-CoV-2-Encoded svRNAs

Viral-derived sncRNAs are also an important family of sncRNAs (Tycowski et al., 2015). To investigate whether SARS-CoV-2-encoded svRNAs are produced in the context of SARS-CoV-2 infection, the seq data were also aligned to the complete genome sequence of SARS-CoV-2 isolate Wuhan-Hu-1 (NC_045512.2). Several SARS-CoV-2-derived svRNAs were identified in SARS-CoV-2 positive samples. The eight most abundant SARS-CoV-2-encoded svRNAs are listed in Table 6.

**TABLE 6:** SARS-CoV-2-encoded svRNAs.

sv-CoV2 small RNAs	G enome position	Sequence	Length (nt)	Derived ORFs of SARS CoV-2	Raw reads	
					Sample 1	Sample 2
sv-CoV2-346	346–382	CGU​ACG​UGG​CUU​UGG​AGA​CUC​CGU​GGA​GGA​GGU​CUU	36	nsp1	43071	3258
sv-CoV2-2825	2,825–2,861	AAU​GAG​AAG​UGC​UCU​GCC​UAU​ACA​GUU​GAA​CUC​GGU	36	nsp3	13535	4426
sv-CoV2-6286	6,286–6,311	CUG​GUG​UAU​ACG​UUG​UCU​UUG​GAG​C	25	nsp3	11743	2939
sv-CoV2-14728	14,728–14,751	AAG​GAA​GGA​AGU​UCU​GUU​GAA​UU	23	nsp12	2026	2188
sv-CoV2-15954	15,954–15,979	AGG​GGC​CGG​CUG​UUU​UGU​AGA​UGA​U	25	nsp12	10998	1846
sv-CoV2-20364	20,364–20,415	ACA​UCU​ACU​GAU​UGG​ACU​AGC​UAA​ACG​UUU​UAA​GGA​AUC​ACC​UUU​UGA​AUU	51	nsp15	10510	3422
sv-CoV2-21145	21,145–21,171	CUU​GGA​GGU​UCC​GUG​GCU​AUA​AAG​AU	26	nsp16	3134	1394
sv-CoV2-26183	26,183–26,216	AUG​AUG​AAC​CGA​CGA​CGA​CUA​CUA​GCG​UGC​CUU	33	3a	15134	4925

**FIGURE 6:**
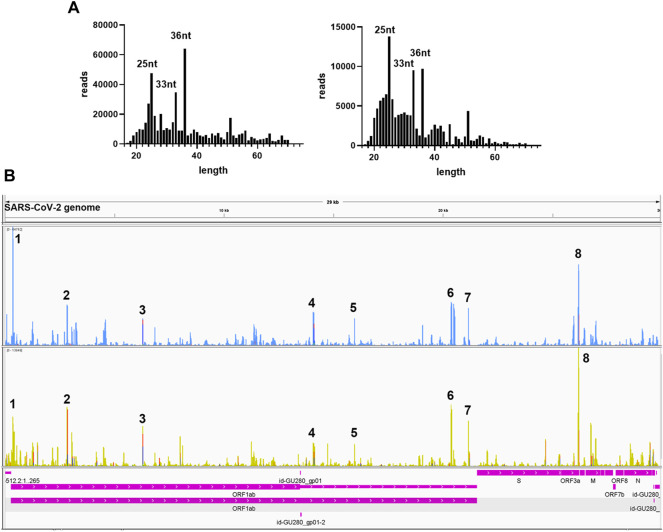
Sequence information of virus-derived sncRNAsA. **(A)** Length distribution of viral small RNAs from two representative patient samples. **(B)** Two representative visual inspections of the small RNA sequences aligning with the SARS-CoV-2 genome. The names of viral genes and the genome positions (nt) are indicated.

We further analyzed the sequences of svRNAs. Since only RNA <200 bp were selected for the cDNA library, our results should not give svRNAs larger than 200 bp. We found that the length of mapped svRNAs ranged from 17 to 75 nt. Interestingly, svRNAs with the length of 25 nt, 33 nt, and 36 nt were enriched (Figure 6A, two representatives are shown). In Figure 6B, the locations of the top 8 svRNAs along the SARS-CoV-2 genome are shown.

### Experimental Validation of SARS-CoV-2-Encoded svRNAs

**FIGURE 7:**
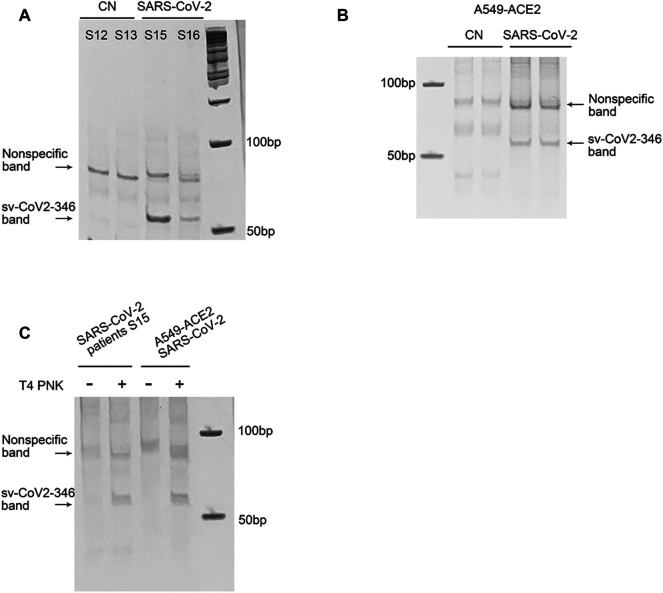
Experimental validation of sv-CoV2-346. **(A)** RT-PCR was performed to detect sv-CoV2-346 in NPS samples of two control (CN, SARS-CoV-2 negative) and two SARS-CoV-2 patients. **(B)** The presence of svCoV2-346 in SARS-CoV-2-infected A549-ACE2 cells. **(C)** Detection of sv-CoV2-346 needs the pretreatment of T4 PNK. The 3′-end of sv-CoV2-346 does not have –OH, as samples without pretreatment of T4 PNK did not result in sv-CoV2-346 bands. All experiments were independently repeated twice.

Among CoV-2-derived svRNAs (sv-CoV2), a 36 nt sv-CoV2, derived from genomic site 346 to site 382 of nsp1 (sv-CoV2-346) had the highest expression. To further validate the presence of sv-CoV2-346, NPS RNAs from two control samples and two COVID-19 samples were treated with T4 PNK and linked to a 3′ RNA linker, and then the RT-PCR was performed. The RT-PCR was also performed without the T4 PNK treatment and RNA linker addition so that the importance of such treatments can be demonstrated. The overall workflow is illustrated in Figures 1B,C. The specific 55 nt RT-PCR products of sv-CoV2-346 were observed in SARS-CoV-2 samples, but not in the control samples, when samples were pretreated with T4 PNK and ligated with an RNA linker (Figure 7A). The length reflected the 36 nt sv-CoV2-346 along with the 3′ RNA linker. In addition, we found that the RT-PCR product of one SARS-CoV-2 sample was more than another one, which was consistent with their read frequency in Seq-data. The presence of sv-CoV2-346 was confirmed in SARS-CoV-2-infected A549-ACE2 cells using RT-PCR (Figure 7B).

Coronavirus-encoded svRNAs have been previously reported to be 18–22 nt long and therefore, share similar lengths with miRNAs (Morales et al., 2017; Cheng et al., 20212021). Coronavirus-encoded svRNAs with lengths longer than 30 nt have not been identified. Herein, we think that the identification of additional SARS-CoV-2-derived svRNAs was benefited from the treatment of T4 PNK and RNA linker ligation at their 3′-end. As shown in Figure 7C, both patient or infected cell samples, without such treatments, did not result in the band presence, supporting the lack of 3′-OH end of sv-CoV2-346 and the necessity of specific T4 PNK treatment for sv-CoV2-346 detection.

**FIGURE 8:**
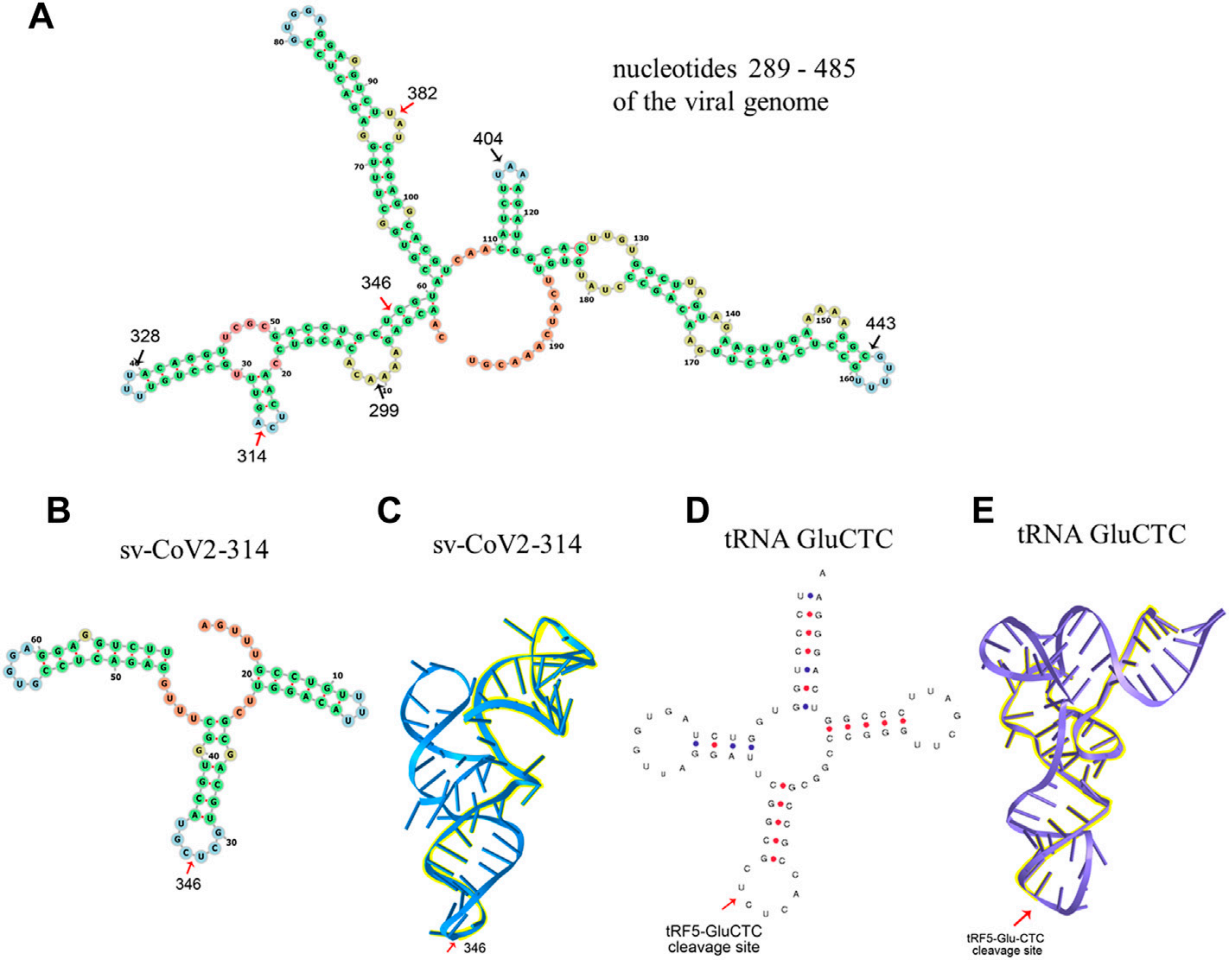
Structure prediction of viral small RNA. **(A)** The secondary structure of viral nucleotides 289–485. The secondary **(B)** and tertiary **(C)** structure of sv-CoV2-314. The secondary **(D)** and tertiary **(E)** structure of a tRNA example GluCTC. Arrows indicated cleavage sites.

Herein, we also initialed to characterize sv-CoV2-346 by predicting the secondary RNA structure of svRNAs. Besides sv-CoV2-346, there were two other svRNAs, sv-CoV2-299 and svCoV2-404, near the region where sv-CoV-346 was derived (Table 7). sv-CoV2-299, sv-CoV2-346, and sv-CoV2-404 were derived from nucleotide 299 to 328, 346 to 382, and 400 to 443, respectively. Therefore, we took the viral genome spanning from 289 to 485, which covers all these three regions with some nt extension on both ends and predicted its RNA secondary structure using RNAfold web server based on minimum free energy to have a clue of biogenesis mechanisms (Hofacker, 2003) (Figure 8A). We found that nucleotides 299, 328, 400, 443, and 382 are all located on loops, implying the cleavage at these five sites along with the single-stranded RNA by ribonuclease (Figure 8A). Only nucleotide 346 was in the middle of the stem (Figure 8A). Interestingly, we found that 68 nt long svRNAs (sv-CoV2-314) overlapped with sv-CoV2-346 (Table 7). We, therefore, took the genome section spanning from 314 to 382 and run the secondary and tertiary structures of sv-CoV2-314 using RNAfold web server and RNAComposer, respectively (Hofacker, 2003; Popenda et al., 2012) (Figures 8B,C). This 68 nt fragment contained three hairpin loops and was folded into an L-shaped-like tertiary structure, and nucleotide 346 was located within the bottom loop (Figures 8B,C). The secondary and tertiary structures of sv-CoV2-314 were similar to tRNA, and nucleotide 346 location was similar to the cleavage site of tRFs (Figures 8D,E). Thus, we speculated that 68 nt sv-CoV2-314 may be the precursor of 36 nt sv-CoV2-346 and the virus may use endonuclease involved in tRF biogenesis to generate viral small RNAs fragments.

**TABLE 7:** RNA sequence information for those with high raw reads and mapping to viral nucleotides 289–485.

sv-CoV2 small RNAs fragments	Genome position	Sequence	Length (nt)	Raw reads	
				Sample 1	Sample 2
sv-CoV2-346	346–382	CGU​ACG​UGG​CUU​UGG​AGA​CUC​CGU​GGA​GGA​GGU​CUU	36	43,071	3,036
sv-CoV2-299	299–328	ACA​CAC​GUC​CAA​CUC​AGU​UUG​CCU​GUU​UU	29	1,931	636
sv-CoV2-404	404–443	AAA​GAU​GGC​ACU​UGU​GGC​UUA​GUA​GAA​GUU​GAA​AAA​GGC	39	1,311	586
sv-CoV2-314	314–382	AGU​UUG​CCU​GUU​UUA​CAG​GUU​CGC​GAC​GUG​CUC​GUA​CGU​GGC​UUU​GGA​GAC​UCC​GUG​G AGGAGGUCUU	68	126	13

## Discussion

In this study, we identified the change in sncRNA expression by SARS-CoV-2. Among sncRNAs, miRNAs have been getting lots of attention in the studies (Vaz et al., 2010; Plieskatt et al., 2014; Max et al., 2018; Hou and Yao, 2021). Standard barcode-ligation-based small RNA-seq are usually designed to capture miRNAs, which usually have 3′-OH ends (Hafner et al., 2008). It is increasingly acknowledged that the 3′-ends of other types of sncRNAs are heterogeneous (Honda et al., 2015), resulting in unsuccessful sequencing barcode ligation in the standard small RNA-seq. In our study, T4 PNK-RNA-seq was employed to profile sncRNAs with heterogeneous ends. Sequencing data revealed that piRNAs and tRFs had higher global expression than miRNAs in NPS (Figure 2A). sncRNAs may carry various unidentified modifications, which are insensitive to T4 PNK treatment. Therefore, T4 PNK-RNA-seq may leave some SARS-CoV-2-impacted sncRNAs unidentified. Herein, the consistency among the seq data, qRT-PCR result, and NB data of tRF5-GluCTC suggested the reliability of T4 PNK-RNA-seq for tRF5 detection.

Notable, among differently expressed tRF5s with mean of normalized counts >20 in control (CN) or SARS-CoV-2 groups (Table 5), we found four tRF5s: tRF5-GluTTC-1-2, tRF5-GlyCCC-6-1, tRF5-LysCTT61 and tRF5-SecTCA-2-1, were not classic tRF5s. While their 3′-ends commonly stop around the anticodon region like classical tRF5s, they lack the first 10-15 nt of the tRNA 5′end. Since they span the complete region of the D loop and the first half of the anticodon loop, we subgrouped and named them as tRF5DC (Supplementary Figure S2). Interestingly, tRF5DC and classic tRF5s were derived from the different tRNA isoacceptors tRNA, suggesting different biogenesis mechanisms of these two type tRFs.

This study further supported that tRFs induction is virus-specific. Previously, we and others have shown that RSV, hepatitis B virus (HBV), and hepatitis C virus (HCV) infections lead to different tRF profile changes (Wang et al., 2013; Selitsky et al., 2015; Zhou et al., 2017; Choi et al., 2020). Compared with tRF induction by RSV, we found that SARS-CoV-2 could induce tRF5-ValCAC, while RSV cannot. On the other hand, we found that tRF5-GlyGCC, which is significantly inducible by RSV, was not induced by SARS-CoV-2. The virus-specific changes in tRFs suggest them to be promising biomarkers for viral infections.

Other than host-derived sncRNAs, sncRNAs can also be derived from viruses. svRNAs have been reported to be involved in the regulation of viral replication, viral persistence, host immune evasion, and cellular transformation (Tycowski et al., 2015). SARS-CoV-2-encoded sncRNAs have been demonstrated by two independent groups (Cheng et al., 20212021; Fu et al., 2021). Using T4-PNK-RNA-seq, several novel svRNAs in SARS-CoV-2 NPS samples were identified. One of them, sv-CoV2-346, was verified to be present in SARS-CoV-2-infected A549-ACE2 cells as well. Due to the limited NPS RNA samples, the leftover RNAs after sequencing were not enough for stem-loop qRT-PCR to validate svRNAs. Thus, we detected sv-CoV2-346 by RT-PCR using the same cDNA generated by the RT step for the qRT-PCR assays for tRF5s. Our RT-PCR revealed a sv-CoV2-346 specific band around 55 nt and a non-specific band. In the future, we will study the relationship between SARS-CoV-2 svRNAs and viral loads/disease severity.

The most widely studied viral sncRNAs are miRNAs-like svRNAs. Both DNA and RNA viruses could encode miRNAs-like svRNAs *via* Dicer-dependent miRNAs biogenesis pathways (Tycowski et al., 2015; Grundhoff and Sullivan, 2011). Among RNA viruses, cytoplasmic restricted RNA viruses were thought incapable of producing miRNA-like svRNAs. However, accumulating evidence indicates cytoplasmic RNA viruses, such as H5N1 influenza virus, enterovirus 71(EV71), West Nile virus (WNV), SARS-CoV, and SARS-CoV-2, also encode viral miRNAs (Morales et al., 2017; Cheng et al., 20212021; Fu et al., 2021; Perez et al., 2010; Li et al., 2018; Weng et al., 2014; Hussain et al., 2012). These cytoplasmic RNA viruses generate viral miRNAs *via* multiple non-canonical miRNAs biogenesis mechanisms. Dicer, not Drosha, is involved in WNV and EV71 viral miRNAs generation (Weng et al., 2014; Hussain et al., 2012). H5N1 influenza virus and SARS-CoV encode viral miRNAs in a Dicer-and Drosha-independent way (Morales et al., 2017; Li et al., 2018). Besides viral miRNAs, the induction of functional svRNAs, which do not look like miRNAs, was reported for cytoplasmic RNA viruses (Perez et al., 2010). However, the knowledge on how cytoplasmic RNA viruses produce svRNAs is limited. One of the interesting observations of newly discovered sv-CoV2 is that sv-CoV2-314 may have a similar tertiary structure as tRNAs and function as the potential precursor of 36 nt svCoV2-346 (Figure 8). Whether a tRNA-like shape (three-leafed clover) of svRNAs makes them as prone as tRNAs to the cleavage during SARS-CoV-2 infection will be investigated in the future. Recently, the cleavage of tRNAs has been reported to be regulated by nt modifications and tRNA anticodon loop is enriched with modification (Blanco et al., 2014; Ranjan and Rodnina, 2016). Therefore, it is possible that the anticodon and/or D loop experience nt modification changes in SARS-CoV-2 infection, resulting in the cleavage. It has been also previously reported that the cleavage of tRNAs is mediated by specific ribonuclease(s) (Lee et al., 2009; Wang et al., 2013; Zhou et al., 2017; Choi et al., 2020). Therefore, it is also possible SARS-CoV-2 favors the activation of certain enzymes to enrich the corresponding sncRNA population. How viruses use the host proteins to control the biogenesis of sncRNAs is still unclear and awaits investigation.

In summary, we investigated COVID-19-impacted sncRNAs comprehensively using the NPS samples by T4 PNK-RNA-seq and modified qRT-PCR method. We are aware that our study has some limitations. For example, T4 PNK-RNA-seq may not catch all types of sncRNAs. In addition, our NPS samples were all from outpatient clinics, which set the limitation to study the correlation of tRF changes with the disease severity. We are closely working with our Molecular Diagnosis Laboratory to obtain the samples from outpatient, inpatient, and ICU services so that whether tRFs serve as disease biomarkers can be determined. Our recent publication on the correlation of tRF changes with Alzheimer’s disease severity supports tRFs to be promising disease biomarkers (Wu et al., 2021). Other than biomarkers, the studies of tRFs in viral infections may also provide insight into therapeutic/prophylactic strategy development. In RSV infection, we found some induced tRFs promote viral replication (Wang et al., 2013; Zhou et al., 2017; Choi et al., 2020). Therefore, any mechanisms associated with their biogenesis and function would not only reveal potential targets to control viral replication but also benefit the ncRNA research community.

## Data Availability

The datasets presented in this study can be found in online repositories. The names of the repository/repositories and accession number(s) can be found below: Gene Expression Omnibus accession number: GSE193555.

## Supplementary Material

Supplementary Figure 1 The pipeline of T4 PNK-RNA-seq and data analyses. **(A)** The flowchart of T4 PNK-RNA-seq. **(B)** The flowchart of the sequencing data analyses.

Supplementary Figure 2 Biogenesis of tRF5s and tRF5DC. tRF5s are generated by the cleavage on the anticodon loop (black arrows); tRF5DCs are derived from the cleavage on the D arm (red or blue arrow) and anticodon loop (black arrows).
