# Supplementary material for: Changes of Small Non-coding RNAs by Severe Acute Respiratory Syndrome Coronavirus 2 Infection
Source: Front Mol Biosci. 2022 Feb 23;9:821137. doi: 10.3389/fmolb.2022.821137 (PMC8905365; doi:10.3389/fmolb.2022.821137)
Supplement: Supplementary file 1 [file Table1.pdf]

Supplementary Table I. The top 10 piRNAs (abundance)

	baseMean[1]
piR-31038	13119.37
piR-38581	5149.83
piR-38756	3153.08
piR-55891	2726.51
piR-33382	2348.61
piR-59293	2231.93
piR-59425	1488.54
piR-58707	1416.84
piR-51761	1292.06
piR-33043	1249.65

[1]baseMean: DESeq2 results of the average of the normalized count values, dividing by size factors, taken over all samples.
