# Supplementary material for: Changes of Small Non-coding RNAs by Severe Acute Respiratory Syndrome Coronavirus 2 Infection
Source: Front Mol Biosci. 2022 Feb 23;9:821137. doi: 10.3389/fmolb.2022.821137 (PMC8905365; doi:10.3389/fmolb.2022.821137)
Supplement: Supplementary file 2 [file Table2.pdf]

Supplementary Table II. The Top 10 tRFs (abundance)

	baseMean[1]	sequence
tRF5-Glu-CTC-2-1	45637.69747	UCCCUGGUGGUCUAGUGGUUAGGAUUCGGCGCU
tRF5-Gly-GCC-3-1	4785.065821	GCAUUGGUGGUUCAGUGGUAGAAUUCUCGCC
tRF5-Glu-TTC-8-1	888.8149209	UCCCCUGUGGUCUAGUGGUUAGGAUUCGGCGCU
tRF5-Gly-GCC-1-5	750.6580236	GCAUGGGUGGUUCAGUGGUAGAAUUCUCGCC
tRF5-Val-CAC-chr1-93	467.6325707	GUUUCCGUAGUGUAGUGGUUAUCACGUUCGCU
tRF5-nm-Tyr-GTA-chr14-8	398.5972534	GCUGAGUGAAGCAUUGGACUGUAA
tRF5-Lys-CTT-2-5	354.265456	GCCCGGCUAGCUCAGUCGGUAGAGCAUGAGACU
tRF5-SeC-TCA-2-1	325.3529339	AGUGGUCUGGGGUGC
tRF5-Glu-TTC-chr1-138	270.7106895	UCCCUGGUGGUCUAGUGGCUAGGAUUCGGCGCU
tRF5-His-GTG-1-8	194.8375191	GGCCGUGAUCGUAUAGUGGUUAGUACUCUGCGUU

[1]baseMean: DESeq2 results of the average of the normalized count values,
dividing by size factors, taken over all samples.
