# Supplementary figures and images for: Changes of Small Non-coding RNAs by Severe Acute Respiratory Syndrome Coronavirus 2 Infection
Source: Front Mol Biosci. 2022 Feb 23;9:821137. doi: 10.3389/fmolb.2022.821137 (PMC8905365; doi:10.3389/fmolb.2022.821137)

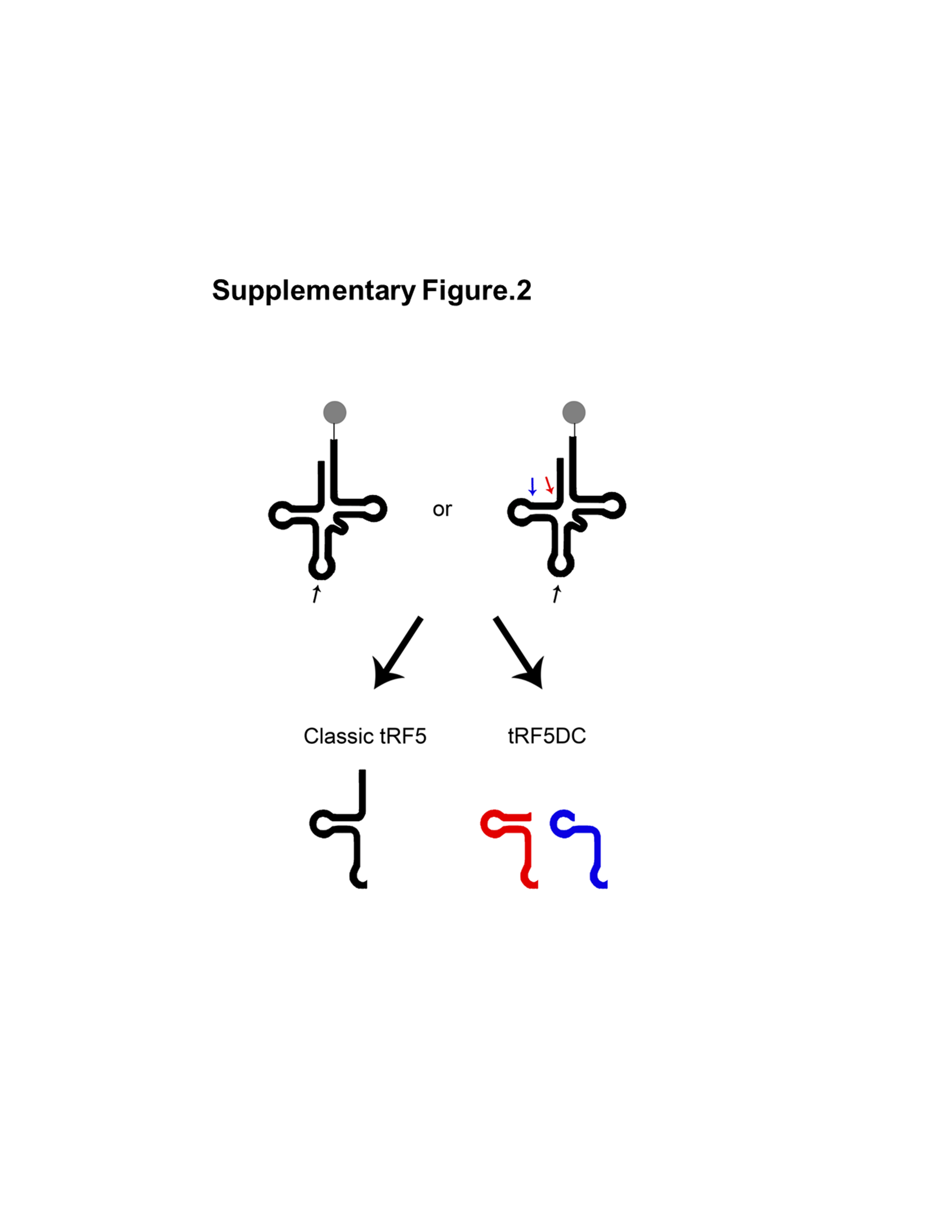

Supplement: Supplementary file 3 [file Image2.TIF]

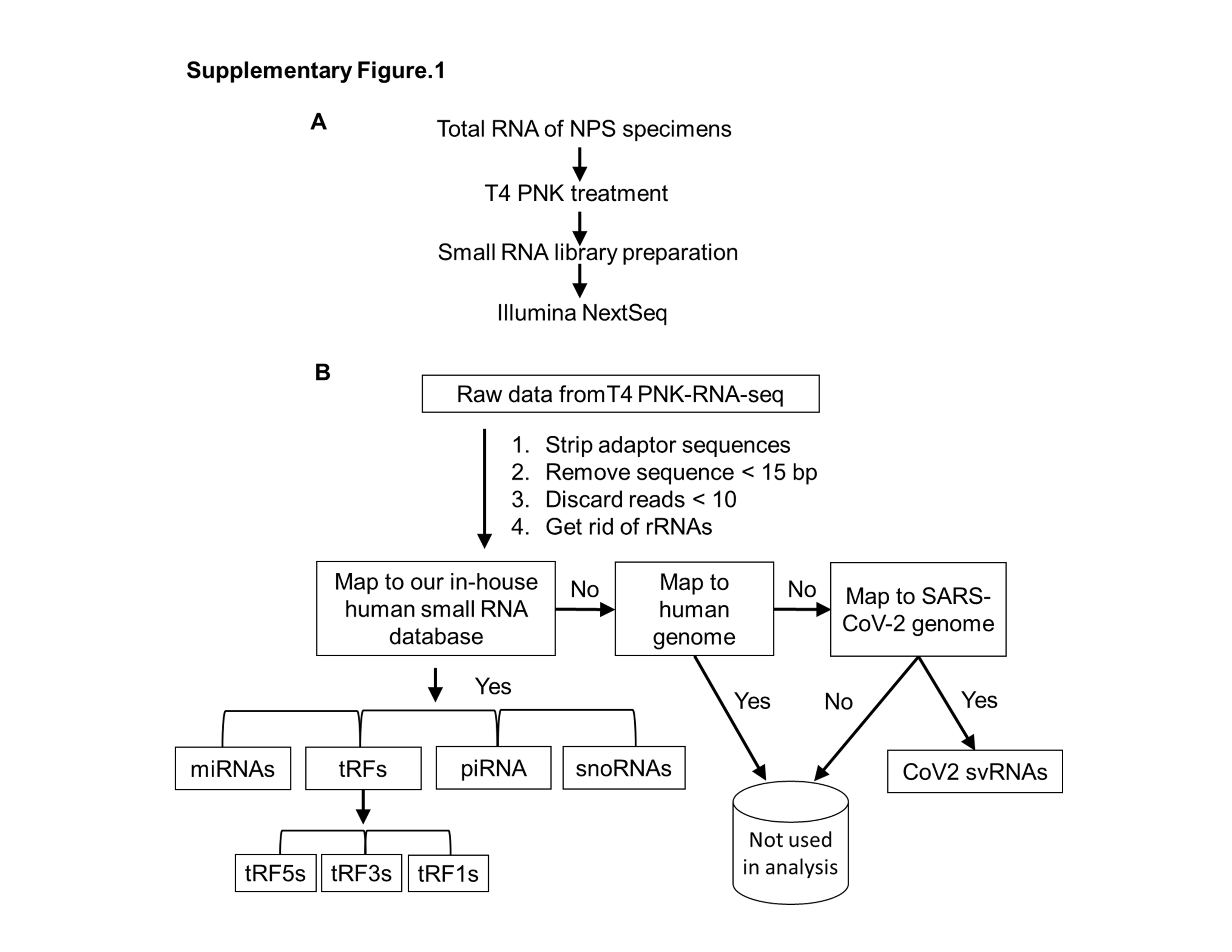

Supplement: Supplementary file 4 [file Image1.TIF]
